# Rho GTPases are involved in S1P‐enhanced glomerular endothelial cells activation with anti‐myeloperoxidase antibody positive IgG

**DOI:** 10.1111/jcmm.13736

**Published:** 2018-07-11

**Authors:** Xiao‐Jing Sun, Min Chen, Ming‐Hui Zhao

**Affiliations:** ^1^ Renal Division Department of Medicine Peking University First Hospital Beijing China; ^2^ Peking University Institute of Nephrology Beijing China; ^3^ Key Laboratory of Renal Disease Ministry of Health of China Beijing China; ^4^ Key Laboratory of Chronic Kidney Disease Prevention and Treatment (Peking University) Ministry of Education Beijing China; ^5^ Peking‐Tsinghua Center for Life Sciences Beijing China

**Keywords:** ANCA, Rho GTPases, sphingosine‐1‐phosphate, vasculitis

## Abstract

Sphingosine‐1‐phosphate (S1P) is a crucial regulator in vascular inflammation. Our recent study found that under pathophysiological concentration in active anti‐neutrophil cytoplasmic antibody (ANCA)‐associated vasculitis (AAV), S1P participated in MPO‐ANCA‐positive IgG‐induced glomerular endothelial cell (GEnC) activation via a S1P receptor (S1PR)‐dependent way. However, the downstream signalling pathways are not fully clear yet. In this study, we demonstrated that Rho guanosine triphosphatases (GTPases) signalling pathways, RhoA and Rac1 in particular, were implicated in MPO‐ANCA‐positive IgG‐mediated GEnCs activation enhanced by pathophysiological concentration of S1P in AAV. These results provide mechanistic insights into vascular barrier dysfunction in AAV, which may facilitate the development of effective therapies.

## 1 INTRODUCTION

Anti‐neutrophil cytoplasmic antibody (ANCA)‐associated vasculitis (AAV) consisted of granulomatosis with polyangiitis (GPA), eosinophilic granulomatosis with polyangiitis (EGPA) and microscopic polyangiitis (MPA).^1^ AAV is characterized by pauci‐immune necrotizing small vessel vasculitis which principally involves glomerular endothelial cells (GEnCs) injury, and circulating autoantibodies against myeloperoxidase (MPO) as well as proteinase 3 (PR3).^2^, ^3^ MPO‐ANCA was reported to induce GEnC activation directly in AAV.^4^

The major sphingolipid metabolite, sphingosine‐1‐phosphate (S1P), is an important regulator in vascular inflammation. S1P and its five G‐protein‐coupled receptors (GPCRs), S1PR1‐5, are implicated in diverse vascular inflammatory conditions.^5^ Our recent studies reported that the circulating levels of S1P and the renal expression of S1PRs were associated with disease activity and renal involvement of AAV,^6^ and S1P participated in GEnC activation induced by MPO‐ANCA‐positive immunoglobulin (Ig)G *via* S1PR2‐5.^7^ However, the downstream signalling pathways of S1P in this process are still not fully clear.

Rho guanosine triphosphatases (GTPases) mediate diverse biological responses including morphogenesis, chemotaxis and cell cycle progression.^8^ It was reported that Rho GTPases, especially Rac1 and RhoA, could regulate endothelial barrier function in response to S1P and its receptors. S1P of physiological level causes the activation of S1PR1, resulting in protection of the endothelial barrier function by inducing the activation of the Rac1 signalling pathway, whereas excessive S1P will bind to S1PR2/S1PR3, leading to the activation of RhoA as well as the disruption of endothelial barrier function.^9^, ^10^, ^11^ In view of the potential role of Rho GTPases in regulating endothelial barrier function, we hypothesized that Rho GTPases, RhoA and Rac1 in particular, might contribute to S1P‐enhanced GEnC activation in the presence of MPO‐ANCA‐positive IgG.

## 2 MATERIALS AND METHODS

### 2.1 Reagents

See the “Supporting Information Data S1.”

### 2.2 Cell culture

Primary human glomerular endothelial cells (GEnC; ScienCell, San Diego, CA, USA) were cultured according to the manufacturer's instructions.

### 2.3 IgG preparation

MPO‐ANCA‐positive IgGs and normal IgGs were prepared as previously described^7^ (detailed in the “Supporting Information Data S1”).

### 2.4 Measurement of Rho GTPase activation

Rac1 and RhoA activation assays were performed following the manufacturer's instructions (Cytoskeleton, Denver, CO, USA).

### 2.5 Measurement of GEnC activation

As biomarkers of endothelial cell activation, levels of soluble vascular cell adhesion molecule‐1(sVCAM‐1) and intercellular adhesion molecule‐1 (sICAM‐1) in the GEnC supernatants were tested with commercially available ELISA kits (R&D, Minneapolis, MN, USA).^12^

### 2.6 Statistical analysis

The normality of our data was evaluated by skewness and kurtosis (both the absolute values were less than 3). Differences were considered statistically significant if *P *<* *.05 (detailed in the “Supporting Information Data S1”).

## 3 RESULTS

### 3.1 Activation of Rho GTPases in MPO‐ANCA‐positive IgG‐treated GEnCs upon stimulation by S1P with pathophysiological concentration in active AAV

**Figure 1:**
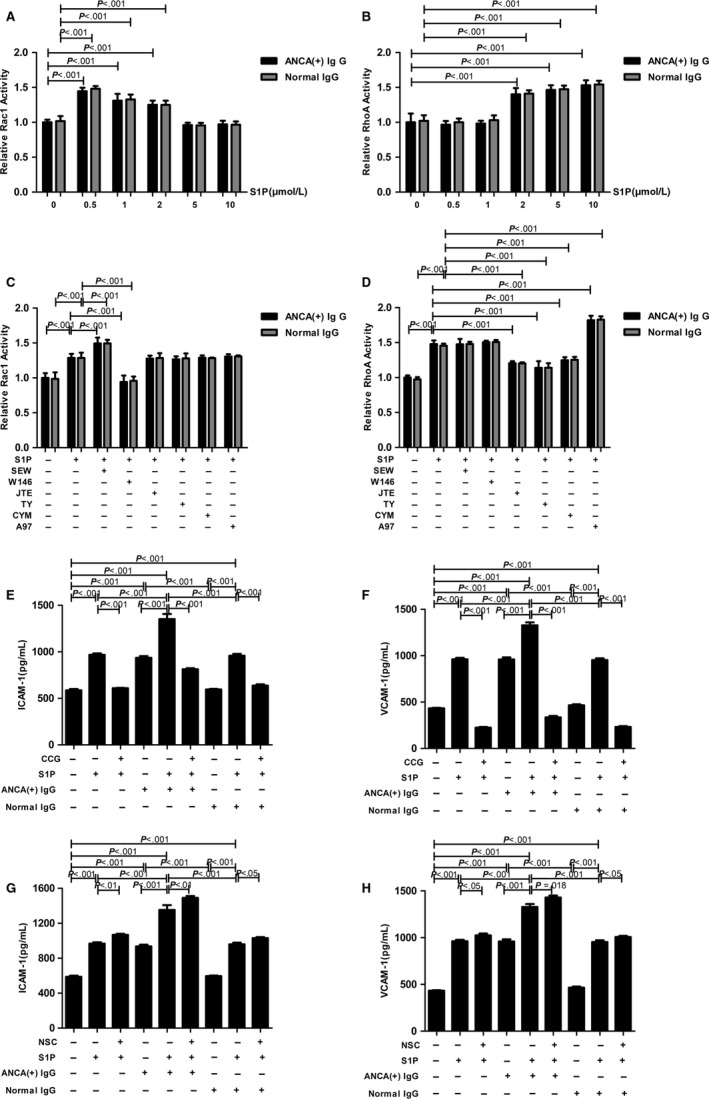
RhoA and Rac1 signalling pathways were implicated in S1P‐enhanced GEnCs activation with MPO‐ANCA‐positive IgG. A, Activation of Rac1 in MPO‐ANCA‐positive IgG‐treated GEnCs upon stimulation by different concentrations of S1P. B, Activation of RhoA in MPO‐ANCA‐positive IgG‐treated GEnCs upon stimulation by different concentrations of S1P. C, The S1PR1‐dependent activation of Rac1 in GEnCs stimulated by S1P plus MPO‐ANCA‐positive IgG. D, The S1PR2‐5‐dependent activation of RhoA in GEnCs stimulated by S1P plus MPO‐ANCA‐positive IgG. E, RhoA antagonist CCG significantly down‐regulated ICAM‐1 level in the supernatants of GEnC stimulated by S1P plus MPO‐ANCA‐positive IgG. F, RhoA antagonist CCG significantly down‐regulated VCAM‐1 level in the supernatants of GEnC stimulated by S1P plus MPO‐ANCA‐positive IgG. G, Rac1 antagonist NSC significantly up‐regulated ICAM‐1 level in the supernatants of GEnC stimulated by S1P plus MPO‐ANCA‐positive IgG. H, Rac1 antagonist NSC significantly up‐regulated VCAM‐1 level in the supernatants of GEnC stimulated by S1P plus MPO‐ANCA‐positive IgG. For the experimental groups which require IgGs, bars represent mean ± SD of MPO‐ANCA‐positive IgGs from plasma of 5 active MPO‐ANCA‐positive primary small vessel vasculitis patients or normal IgGs from plasma of five healthy donors individually. As for the experimental groups which did not require IgGs (eg, GEnCs treated with S1P alone), bars represent mean ± SD of repeated measurements of five independent experiments

Rac1 and RhoA activity were determined after MPO‐ANCA‐positive IgG or normal IgG‐treated GEnCs were stimulated by S1P at gradient concentrations. We found that compared with GEnCs treated with solely MPO‐ANCA‐positive IgG, the activity of Rac1 in MPO‐ANCA‐positive IgG‐treated GEnCs increased significantly at low concentration range of 0.5 and 1 μmol/L S1P; whereas in the presence of MPO‐ANCA‐positive IgG, the activity of RhoA increased significantly at high concentration range of 5 and 10 μmol/L S1P in GEnCs. It is noteworthy that 2 μmol/L S1P, which was comparable to the levels of circulating S1P in active AAV patients,^7^ significantly increased the activity of both Rac1 and RhoA in MPO‐ANCA‐positive IgG‐treated GEnCs compared with those treated with MPO‐ANCA‐positive IgG alone (expressed as percentages of the control: 125.2 ± 5.9% vs 100%, *P *<* *.001 by ANOVA; 140.1 ± 8.9% vs 100%, *P *<* *.001 by ANOVA) (Figure 1A,B). Rac1 or RhoA activity showed no significant difference between the MPO‐ANCA‐positive IgG group and normal IgG group. However, compared with normal IgG, MPO‐ANCA‐positive IgG significantly increased the levels of ICAM‐1 and VCAM‐1 in the supernatants of GEnCs stimulated by 2 μmol/L S1P (Figure S1,S2,S3).

### 3.2 The S1PR‐dependent activation of Rho GTPases in GEnCs stimulated by S1P plus MPO‐ANCA‐positive IgG

Pre‐incubation of GEnCs with the S1PR1 selective agonist SEW could significantly increase the activity of Rac1 in GEnCs treated by S1P plus MPO‐ANCA‐positive IgG, with the increase rate of 16.2 ± 6.5%, while the inhibition of S1PR1 with the antagonist W146 attenuated the Rac1 activity in GEnCs treated by S1P combined with MPO‐ANCA‐positive IgG by 26.9 ± 7.3%. On the contrary, compared with that without the antagonist, the S1PR2‐4 selective antagonists JTE, TY and CYM significantly down‐regulated the relative activity of RhoA in GEnCs treated by S1P plus MPO‐ANCA‐positive IgG (expressed as percentages of the control, 120.5 ± 2.9% vs 147.9 ± 5.2%, *P *<* *.001 by ANOVA; 113.9 ± 9.4% vs 147.9 ± 5.2%, *P *<* *.001 by ANOVA; 124.7 ± 4.4% vs 147.9 ± 5.2%, *P *<* *.001 by ANOVA, respectively), while the S1PR5 agonist A97 up‐regulated the RhoA activity (181.9 ± 6.6% vs 147.9 ± 5.2%, *P *<* *.001 by ANOVA, with the increase rate of 23.0 ± 4.5%) (Figure 1C,D). These results indicated that S1P, with pathophysiological concentration of active AAV patients, induced the activation of Rac1 and RhoA *via* S1PR1 and S1PR2‐5 in GEnCs in the presence of MPO‐ANCA‐positive IgG, respectively.

### 3.3 The effect of Rac1 or RhoA on S1P‐induced ICAM‐1/VCAM‐1 expression of GEnCs in the presence of MPO‐ANCA‐positive IgG

Pre‐incubation of GEnCs with the RhoA antagonist CCG significantly decreased ICAM‐1 and VCAM‐1 levels in the supernatants of GEnCs stimulated by S1P plus MPO‐ANCA‐positive IgG (1352.33 ±  122.73 pg/mL vs 812.91 ± 25.12 pg/mL, *P *<* *.001 by ANOVA; 1328.41 ± 69.02 pg/mL vs 336.13 ± 31.64 pg/mL, *P *<* *.001 by ANOVA, respectively). By contrast, the ICAM‐1 and VCAM‐1 levels in the supernatants of GEnCs stimulated by S1P combined with MPO‐ANCA‐positive IgG increased significantly upon pre‐incubation with Rac1 antagonist NSC (1352.33 ± 122.73 pg/mL vs 1490.04 ±  46.28 pg/mL, *P *<* *.01 by ANOVA; 1328.41 ± 69.02 pg/mL vs 1429.28 ± 46.54 pg/mL, *P *=* *.018 by ANOVA, respectively) (Figure 1E‐H). Collectively, RhoA signalling pathway dominated S1P‐induced ICAM‐1/VCAM‐1 up‐regulation of MPO‐ANCA‐positive IgG‐treated GEnCs, whereas Rac1 signalling pathway exerted opposite effect during this process.

## 4 DISCUSSION

**Figure 2:**
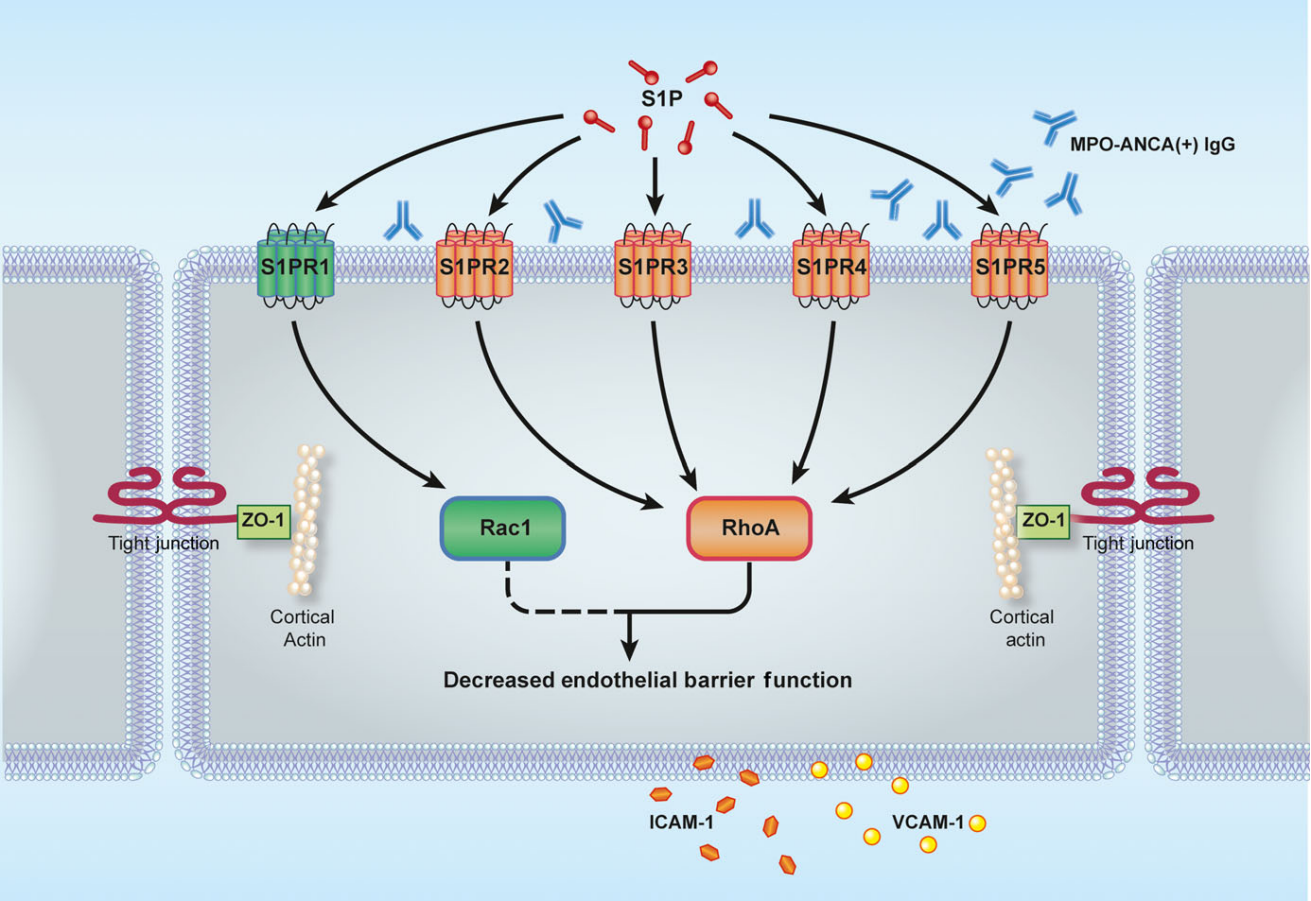
Proposed working model for the role of Rho GTPases in S1P‐induced GEnC activation in the presence of MPO‐ANCA‐positive IgG. Under pathophysiological concentration of S1P in active AAV patients, the activation of S1PR2‐5 and their downstream RhoA signalling pathway dominates the S1P‐induced MPO‐ANCA‐positive IgG‐mediated endothelial activation, whereas the activation of S1PR1 and Rac1 signalling pathway exerts opposite effect during this process. The imbalance between different S1PRs and Rho GTPases activation might participate in the development of AAV. S1P, sphingosine‐1‐phosphate; S1PR, sphingosine‐1‐phosphate receptor; ICAM‐1, intercellular cell adhesion molecule‐1; VCAM‐1, vascular cell adhesion molecule‐1; ZO‐1, zonula occluden‐1

In our present study, we demonstrated that under pathophysiological concentration in active AAV patients, S1P could activate both RhoA and Rac1 signalling pathways in MPO‐ANCA‐positive IgG‐treated GEnCs. According to Singleton et al, RhoA and Rac1 play opposing roles in regulating endothelial barrier function in response to differential activation of S1PRs.^11^ RhoA activated by S1PR2/3 disrupts endothelial barrier function by enhancing the formation of contractile stress fibres which connect to junctions and generate pulling forces within neighbouring cells, therefore inducing destabilization of cell contact and internalization of molecules in tight junctions and adherent junctions.^13^ Loss of endothelial cell‐cell contact and increased permeability also facilitates leukocyte transendothelial migration and damage to endothelium, which is of vital importance in AAV.^14^ Contrary to RhoA, Rac1 activated by S1PR1 enhances endothelial barrier function by inducing reorganization of the actin cytoskeleton as well as affecting the formation of lamellipodia and membrane ruffles.^15^ In the present study, we found that RhoA activated by S1PR2‐5 dominated the S1P‐induced ICAM‐1 and VCAM‐1 up‐regulation of GEnCs in the presence of MPO‐ANCA‐positive IgG, while Rac1 activated by S1PR1 exerted opposite effect during this process, suggesting that the imbalance between RhoA and Rac1 signalling pathways might contribute to GEnC activation in the presence of MPO‐ANCA‐positive IgG. Thus, the final barrier regulating efficacy of S1P might depend on the balance of the expression and activation of different S1P receptors and their distinct downstream Rho GTPase signalling pathways in GEnCs (Figure 2).

In conclusion, our current study demonstrated that Rho GTPases signalling pathways were involved in S1P‐enhanced GEnCs activation with MPO‐ANCA‐positive IgG. RhoA activated by S1PR2‐5 dominated the S1P‐induced GEnC activation in the presence of MPO‐ANCA‐positive IgG, while Rac1 activated by S1PR1 exerted opposite effect during this process. These findings provide us with more clues to determine the role of and Rho GTPases and S1P in the development of AAV.

## CONFLICT OF INTEREST

No conflict of interest to declare.
