# Supplementary figures and images for: Rho GTPases are involved in S1P‐enhanced glomerular endothelial cells activation with anti‐myeloperoxidase antibody positive IgG
Source: J Cell Mol Med. 2018 Jul 11;22(9):4550–4. doi: 10.1111/jcmm.13736 (PMC6111853; doi:10.1111/jcmm.13736)

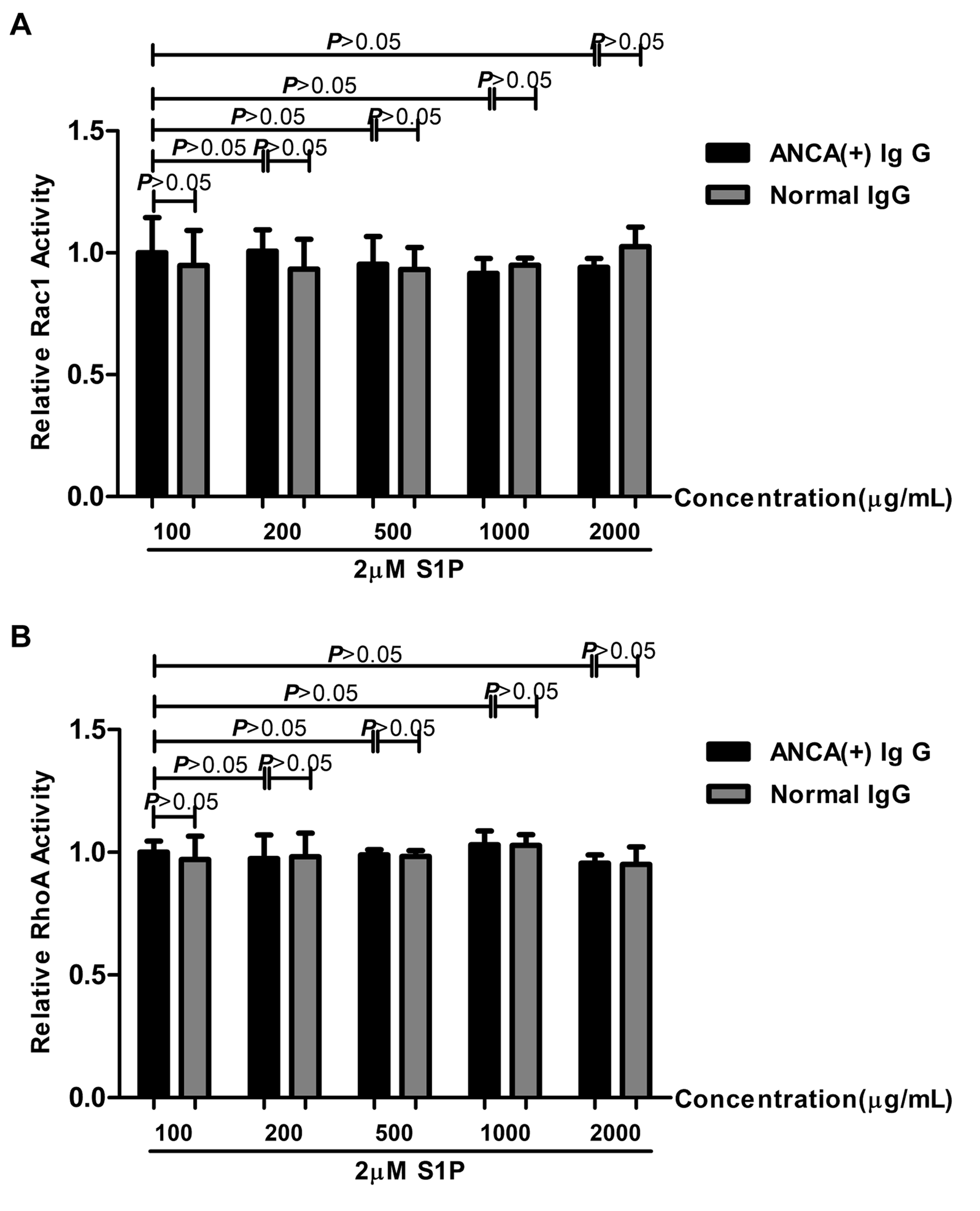

Supplement: Supplementary file 1 [file JCMM-22-4550-s001.tif]

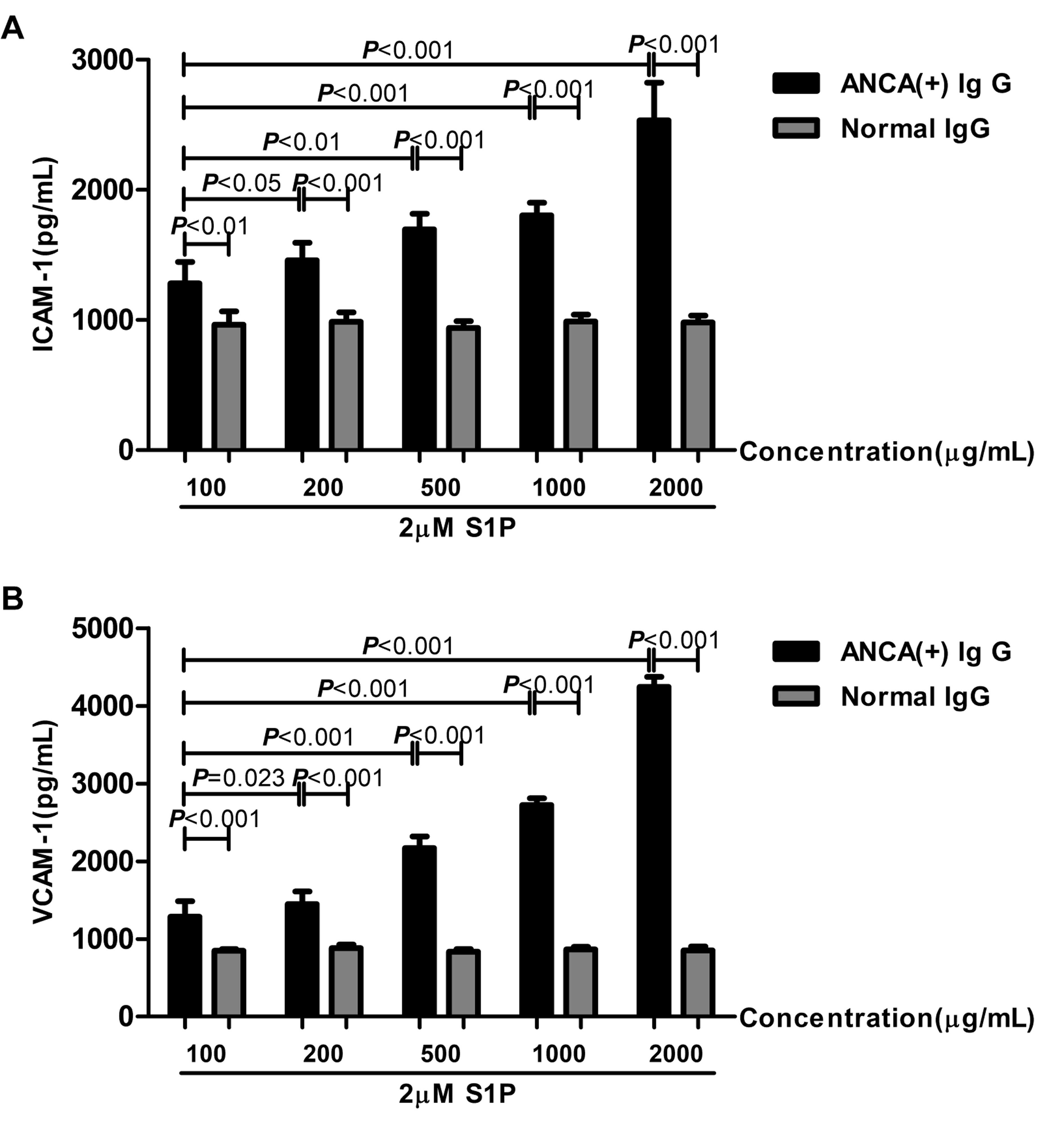

Supplement: Supplementary file 2 [file JCMM-22-4550-s002.tif]

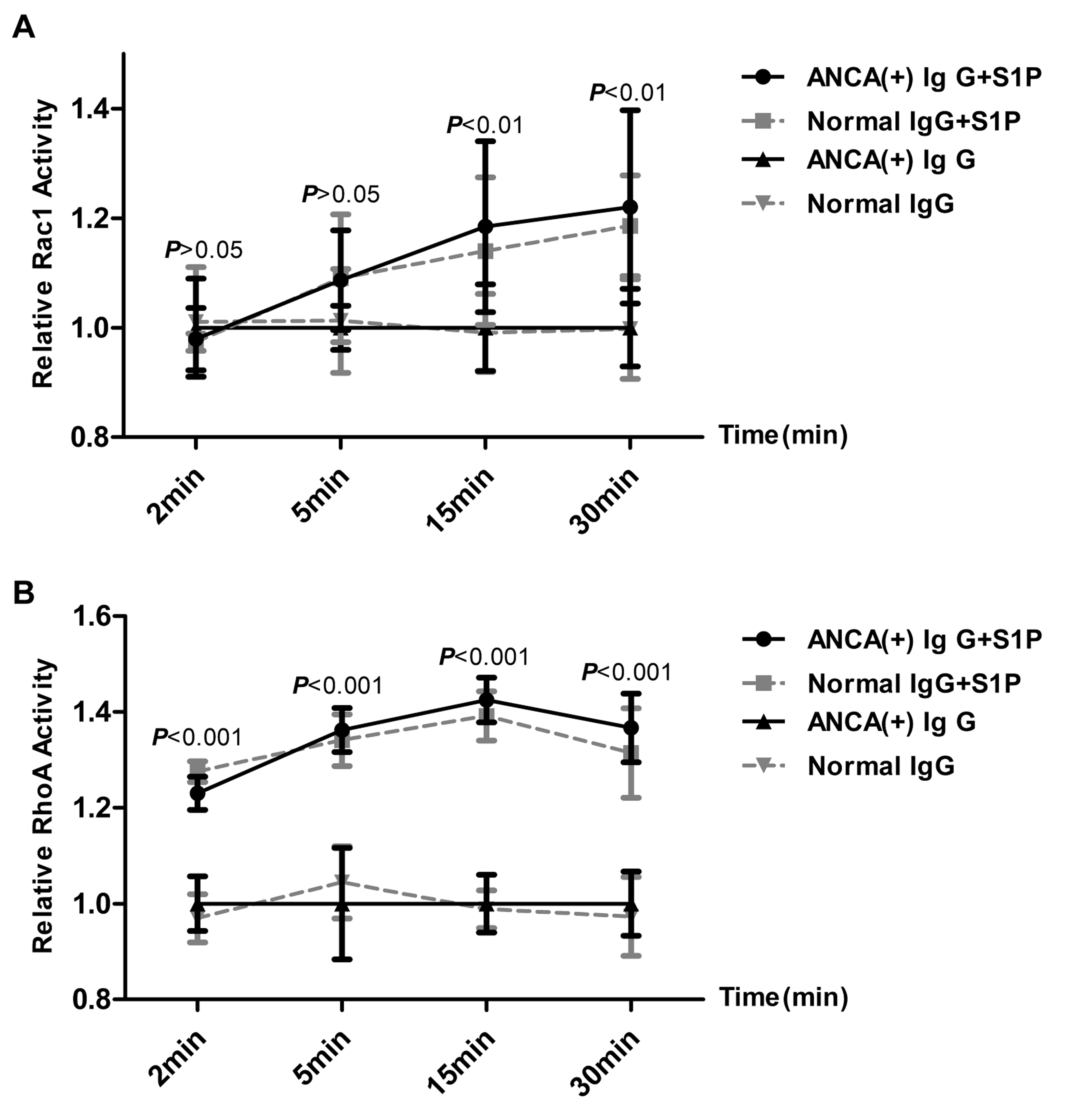

Supplement: Supplementary file 3 [file JCMM-22-4550-s003.tif]
