# Supplementary material for: Rho GTPases are involved in S1P‐enhanced glomerular endothelial cells activation with anti‐myeloperoxidase antibody positive IgG
Source: J Cell Mol Med. 2018 Jul 11;22(9):4550–4. doi: 10.1111/jcmm.13736 (PMC6111853; doi:10.1111/jcmm.13736)
Supplement: Supplementary file 4 [file JCMM-22-4550-s004.doc]

**Supporting information**

**Materials and methods**

**1. Reagents**

Bioactive sphingolipid metabolite S1P was purchased from Sigma-Aldrich (Darmstadt, Germany). Potent and selective agonists and antagonists of S1PR1-5 were obtained from Tocris (Louis, MO, USA). SEW2871 is a specific agonist for S1PR1 [1]. W146, JTE013 (JTE), TY52156 (TY), CYM50358 (CYM) are specific antagonists for S1PR1-4, respectively [2-5]. A971432 (A97) is a specific agonist for S1PR5 [6]. RhoA inhibitor CCG1423 (CCG) and Rac1 inhibitor NSC23766 (NSC) were purchased from Selleck (Houston, TX, USA).

**2. Cell culture**

Primary human glomerular endothelial cells (GEnC; ScienCell, San Diego, CA, USA) were cultured in endothelial cell basal medium (ECM) (ScienCell San Diego, CA, USA) supplemented with 1% endothelial cell growth factor, 1% penicillin/streptomycin and 10% fetal bovine serum (FBS). Cultures were grown in an atmosphere of 5% CO_2_ at 37°C. After starving in ECM with additional 0.5% FBS for 12hrs, GEnCs were washed and stimulated under the following conditions: GEnCs stimulated by MPO-ANCA-positive IgG alone, normal IgG alone, MPO-ANCA-positive IgG plus S1P as well as normal IgG plus S1P. GEnCs cultured in MPO-ANCA-positive IgG served as control. The concentration of S1P was 2μM, which was comparable to the circulating levels of S1P in active AAV patients, as reported in our previous study [7].

**3. IgG preparation**

According to the previously described methods, MPO-ANCA-positive IgGs and normal IgGs were prepared from plasma of five MPO-ANCA positive AAV patients in active stage and five healthy volunteers, respectively [8]. Of the 5 patients with active AAV, 2 (40.0%) were male and 3(60.0%) were female, with an average age of 64.0±5.2 years at diagnosis. All patients were perinuclear ANCA (pANCA)–positive, and these sera all recognized MPO. The level of initial serum creatinine was 549.6±177.0 (range 356.7–800.9) μmol/L (Table S1). Further, we screened the prepared IgGs for the presence of anti-endothelial cell antibody (AECA) [9], and AECA-positive IgGs were excluded in our current study. Our research was approved by the clinical research ethics committee of the Peking University First Hospital and in compliance with the Declaration of Helsinki.

**4. Measurement of Rho GTPase activation**

Rac1 and RhoA activation assays were performed following the manufacturer’s instructions (Cytoskeleton, Denver, CO, USA). The Rho GTPase activation assay kits contain a Rac1 or RhoA-GTP-binding protein which linked to the 96-well plate. Active, GTP-bound Rac1 or RhoA protein in the cell lysates binds to the wells, while inactive GDP-bound Rac1 or RhoA protein is removed in washing steps. The bound active Rac1 or RhoA protein was then detected by a Rac1 or RhoA protein-specific antibody. After incubated with a HRP detection reagent, the reaction was stopped. Eventually we measured the absorbance of the plate at 490 nm. The degree of Rho GTPase activation was determined by comparing the readings of activated cell lysates with those of non-activated cell lysates.

**5. Measurement of GEnC activation**

As biomarkers of endothelial cell activation, levels of soluble vascular cell adhesion molecule-1(VCAM-1) and intercellular adhesion molecule-1 (sICAM-1) in the GEnC supernatants were tested with commercially available ELISA kits (R&D, Minneapolis, MN, USA) [10]. The assays were conducted following the manufacturer’s instructions. In the S1P-induced sICAM-1 expression assay, GEnCs were pre-incubated with the aforementioned Rho GTPase antagonists for different doses and time-points (1μM, 30 minutes; 2μM, 30 minutes; 5μM, 30 minutes; 10μM, 30minutes; 1μM, 1 hour; 2μM, 1 hour; 5μM, 1 hour; 10μM, 1 hour). Eventually 2μM CCG and 5μM NSC at 30 minutes were selected due to the highest inhibition/increase rate.

**6. Statistical analysis**

SPSS version 13.0 was used to perform data analysis (SPSS Inc., Chicago, IL, USA). The normality of our data was evaluated by skewness and kurtosis (both the absolute values were less than 3). Quantitative data were expressed as mean ± standard deviation (for data that were normally distributed), or median and interquartile range (for data that were not normally distributed). Differences in quantitative parameters between groups were assessed using one-way ANOVA analysis followed by LSD multiple-comparison test (for data that were normally distributed) or Kruskal–Wallis test (for data that were not normally distributed), as appropriate. Differences were considered statistically significant if *P*<0.05.

**References**

1. Hale JJ, Lynch CL, Neway W, et al. A rational utilization of high-throughput screening affords selective, orally bioavailable 1-benzyl-3-carboxyazetidine sphingosine-1-phosphate-1 receptor agonists. J Med Chem. 2004;47:6662-5.

2. Sanna MG, Wang SK, Gonzalez-Cabrera PJ, et al. Enhancement of capillary leakage and restoration of lymphocyte egress by a chiral S1P1 antagonist in vivo. Nat Chem Biol. 2006;2:434-41.

3. Inoki I, Takuwa N, Sugimoto N, et al. Negative regulation of endothelial morphogenesis and angiogenesis by S1P2 receptor. Biochem Biophys Res Commun. 2006;346:293-300.

4. Hirata N, Yamada S, Shoda T, et al. Sphingosine-1-phosphate promotes expansion of cancer stem cells via S1PR3 by a ligand-independent Notch activation. Nat Commun. 2014;5:4806.

5. Guerrero M, Urbano M, Velaparthi S, et al. Discovery, design and synthesis of the first reported potent and selective sphingosine-1-phosphate 4 (S1P4) receptor antagonists. Bioorg Med Chem Lett. 2011;21:3632-6.

6. Hobson AD, Harris CM, van der Kam EL, et al. Discovery of A-971432, An Orally Bioavailable Selective Sphingosine-1-Phosphate Receptor 5 (S1P5) Agonist for the Potential Treatment of Neurodegenerative Disorders. J Med Chem. 2015;58:9154-70.

7. Sun XJ, Wang C, Zhang LX, et al. Sphingosine-1-phosphate and its receptors in anti-neutrophil cytoplasmic antibody–associated vasculitis. Nephrol Dial Transplant. 2017;32:1313-22.

8. Schreiber A, Rolle S, Peripelittchenko L, et al. Phosphoinositol 3-kinase-gamma mediates antineutrophil cytoplasmic autoantibody-induced glomerulonephritis. Kidney Int. 2010;77:118-28.

9. Carvalho D, Savage CO, Black CM, et al. IgG antiendothelial cell autoantibodies from scleroderma patients induce leukocyte adhesion to human vascular endothelial cells in vitro. J Clin Invest. 1996;97:111-9.

10. Page AV, Liles WC. Biomarkers of endothelial activation/dysfunction in infectious diseases. Virulence. 2013;4:507-16.

**Figure legends**

**Figure S1.** **Activation of Rac1 and RhoA in GEnCs stimulated by different concentrations of MPO-ANCA-positive IgG and normal IgG in the presence of the pathophysiological concentration of S1P in AAV.**

Rac1 and RhoA activity were determined after 2 μM S1P-treated GEnCs were stimulated by different concentrations of MPO-ANCA-positive IgG and normal IgG. Neither normal IgG nor MPO-ANCA-positive IgG could significantly increase the activity of RhoA or Rac1.

**Figure S2. Levels of ICAM-1 and VCAM-1 in the supernatants of GEnCs stimulated by different concentrations of MPO-ANCA-positive IgG and normal IgG in the presence of the pathophysiological concentration of S1P in AAV**

ICAM-1 and VCAM-1 levels were determined after 2 μM S1P-treated GEnCs were stimulated by different concentrations of MPO-ANCA-positive IgG and normal IgG. Compared with normal IgG, MPO-ANCA-positive IgG could significantly increase the levels of ICAM-1 and VCAM-1 in the supernatants of GEnCs.

**Figure S3. Activation of Rac1 and RhoA in MPO-ANCA-positive IgG-treated GEnCs upon stimulation by the pathophysiological concentration of S1P in AAV for different time points.**

Rac1 and RhoA activation was investigated in GEnCs stimulated by 2μM S1P in the presence of MPO-ANCA-positive IgG for indicated times: 2min, 5min, 15min and 30min, and we found that relative Rac activity showed a significant increase within 2min and reached a peak at 15min, whereas relative RhoA activity increased to the maximum at 30min. No significant difference was observed between the MPO-ANCA-positive IgG group and normal IgG group. Eventually, 2μM S1P at 15min and 30min was chosen for the following Rac1 and RhoA activation assays, respectively, due to the highest increase rate. The p values in this figure refer to the comparison between the groups of GEnCs stimulated by MPO-ANCA-positive IgG alone and the groups of GEnCs treated with MPO-ANCA-positive IgG plus S1P.

**Table S1.** **General data of patients with active AAV**

| Parameters | AAV patients |
| --- | --- |
| General clinical data |  |
| Subjects, no. | 5 |
| Male/female, no. | 2/3 |
| Age at disease onset, Mean ± SD | 64.0±5.2 |
| Serum creatinine, μmoles/liter |  |
| Mean ± SD | 546.9±177.0 |
| Range | 356.7-800.9 |
| Renal insufficiency at diagnosis | 5(100%) |
| Skin rash | 0(0%) |
| Arthralgia | 1(20.0%) |
| Muscle pain | 0(0%) |
| Pulmonary | 3(60.0%) |
| ENT | 1(20.0%) |
| Ophthalmic | 1(20.0%) |
| Gastrointestinal | 2(40.0%) |
| Nervous system | 2(30.0%) |
| BVAS | 26.5±5.0 |
| Pathologic data |  |
| Subjects, no. | 5 |
| Glomerular lesions |  |
| Total crescents | 52.2%±27.1% |
| Cellular crescents | 32.8%±28.1% |
| Tubulointerstitial lesions |  |
| Interstitial infiltration(-/+/++/+++) | 0/0/5/0 |
| Interstitial fibrosis(-/+/++) | 1/0/4 |
| Tubular atrophy(-/+/++) | 0/5/0 |

**[Abbreviations]** AAV= antineutrophil cytoplasmic antibody associated vasculitis; BVAS= Birmingham Vasculitis Activity Score; ENT = ear, nose and throat; SD = standard deviation.
